# Statistical Analysis of Machining Parameters on Burr Formation, Surface Roughness and Energy Consumption during Milling of Aluminium Alloy Al 6061-T6

**DOI:** 10.3390/ma15228065

**Published:** 2022-11-15

**Authors:** Sajid Raza Zaidi, Najam Ul Qadir, Syed Husain Imran Jaffery, Muhammad Ali Khan, Mushtaq Khan, Jana Petru

**Affiliations:** 1 School of Mechanical and Manufacturing Engineering (SMME), National University of Sciences and Technology (NUST), Islamabad 44000, Pakistan; 2 Department of Mechanical Engineering, College of Electrical and Mechanical Engineering (CEME), National University of Sciences and Technology (NUST), Islamabad 44000, Pakistan; 3 Department of Machining, Assembly and Engineering Metrology, Mechanical Engineering Faculty, VŠB-Technical University of Ostrava, 17. Listopadu 2172/15, 708 00 Ostrava, Czech Republic

**Keywords:** milling, aluminum alloy Al 6061-T6, statistical analysis, sustainable machining

## Abstract

Due to the increasing demand for higher production rates in the manufacturing sector, there is a need to manufacture finished or near-finished parts. Burrs and surface roughness are the two most important indicators of the surface quality of any machined parts. In addition to this, there is a constant need to reduce energy consumption during the machining operation in order to reduce the carbon footprint. Milling is one of the most extensively used cutting processes in the manufacturing industry. This research was conducted to investigate the effect of machining parameters on surface roughness, burr width, and specific energy consumption. In the present research, the machining parameters were varied using the Taguchi L9 array design of experiments, and their influence on the response parameters, including specific cutting energy, surface finish, and burr width, was ascertained. The response trends of burr width, energy consumption, and surface roughness with respect to the input parameters were analyzed using the main effect plots. Analysis of variance indicated that the cutting speed has contribution ratios of 55% and 47.98% of the specific cutting energy and burr width on the down-milling side, respectively. On the other hand, the number of inserts was found to be the influential member, with contribution ratios of 68.74% and 35% of the surface roughness and burr width on the up-milling side. The validation of the current design of the experiments was carried out using confirmatory tests in the best and worst conditions of the output parameters.

## 1. Introduction

Milling is an interrupted material removal process in which material is subtracted from a work piece by a rotating tool which may have more than one cutting edge. It is one of the most commonly used machining operations due to its capability to make diverse shapes with better production yield [1,2]. During any milling operation, the variable machining parameters are width and depth of cut, type of lubricant used, cutting tool path, number of cutting edges, tool material, feed rate, tool diameter, cutting speed, and the insert geometry used for machining [3]. With regard to the machining parameters, some of the responses that are subjective include burr formation, surface roughness, energy consumption, tool wear, chip types, and production rate [4,5,6].

During a milling operation, wherever a workpiece and cutting tool come in contact various forms of burrs can be witnessed [7,8,9]. However, in brittle materials, the trend of the burr formation is different from that of the ductile materials [10]. As compared to the down-milling side, generally smaller sized burrs have been reported on the up-milling side of the workpiece. To keep the manufactured part in the limits of geometric tolerance, it is necessary to remove the burrs from the workpiece. As compared to macro milling, the deburring process in micro milling is more essential because at times the burrs are greater in size than the cutting tool [11]. With the increase in the depth of the cut, the cutting speed and feed per tooth decrease in the burr width, as has already been reported in the literature [12]. In another study, it has been reported that larger sized burrs were observed with the increase in spindle speed and tool diameter [13]. Burr size has been reported to be dependent on chip thickness, friction angle [14], residual stresses [15], and tool coatings as well [16]. In addition to this, many efforts have been made to achieve burr-free machining. For this purpose, it has been reported that the exit surface angle of the workpiece plays a key role in burr-free milling [15]. Furthermore, to minimize each form of burr, the optimum machining parameters are different [17]. Nevertheless, the pattern of burr formation with the varying machining parameters is reported to be conflicting [12,18,19]. One of the most significant considerations in dimensional accuracy is the surface roughness [20]. Mechanical properties such as the fatigue life, tensile strength, and surface topography of a workpiece are greatly influenced by the surface roughness [21,22]. The three major factors that influence the surface finish of any machined part are the geometric factors, the work piece material, and the vibrations of the machine tool. Cutting tool geometry, feed rate, and type of machining operation govern the geometric parameters [23]. By employing suitable geometric factors, the surface roughness can be improved [24,25], predicted [26,27,28], and optimised [29]. To achieve the desired surface finish machineability of a workpiece, the work material factor also sets a limitation [1,30]. An improved surface finish is reported by using a higher spindle speed and a reduction in the depth of the cut, the cutting speed, and the feed per tooth [31]. One of the pioneering research works, which introduced the concept of maximum production rate and minimum cost, was presented in 1950 [32]. In addition to machining costs, the energy cost is one of the major considerations which has economic and environmental effects [33,34]. Machining systems are usually less energy efficient [35,36], and their energy efficiency has been reported to be as low as 30% [37,38]. In modern milling machines, the machine tool uses a large amount of energy, and energy utilization during the material process can be as low as 14% of the entire energy utilized by the machine tool [39]. Along with the total energy consumption, specific energy consumption, and machine tool efficiency are also dependent on the machining parameters [40]. By the optimisation of the tool path and by the selection of suitable machining parameters and cutting tools, an excess consumption of 6 to 40% of the energy can be saved [41].

As the material removal rate (MRR) is a function of cutting speed, width of cut, and feed rate, the specific energy consumption therefore cannot be predicted by the material removal rate alone [42]. The amount of energy consumption during any milling operation is dependent on the cutting speed, feed rate, width of cut, and depth of cut [43,44,45,46]. To diminish the negative effects of manufacturing on nature and society, the concept of environmentally conscious manufacturing is gaining importance, with emphasis on the efficient use of natural resources and raw material [47]. Energy consumption can be reduced, and a better energy efficiency can be achieved during milling operations if suitable machining parameters are used. Recently, techniques for the reduction in energy consumption and the achievement of finished or near-finished parts are gaining importance. It has been reported by past researchers multiple times that energy consumption [48,49], surface roughness [50,51], and burr formation [12,17] are greatly influenced by machining parameters, which include machining conditions, cutting speed, feed rate, number of inserts, and depth of cut.

The study of the literature highlights that the improvement in surface finish and the reduction in burr formation and energy consumption can be attained by the careful selection of suitable machining parameters. This will not only lead to sustainable machining and better surface quality and dimensional accuracy but will also be helpful in producing near-finished parts with a minimum need for a deburring process. However, limited research has been published on burr formation during macro milling. Similarly, the literature contains few noteworthy works related to specific cutting energy, which is a significant sustainability index. The number of inserts used during any milling operation also affects the specific energy consumption and surface finish; however, their effect has not been stated in the published literature. This study aims to reduce burr size, surface roughness, and specific cutting energy. The reduction in burr size and surface roughness will be helpful in reducing the production time and in manufacturing the parts with a net or near-net shape. The reduction in specific energy consumption will lead to sustainable machining and a reduction in the carbon footprint. The novelty of the current research is drawn from the above-stated research gaps. In this study, a statistical approach was used to detect the effect of the machining parameters on the specific energy consumption, burr width, and surface roughness. Moreover, the contribution ratio and the significance of each machining parameter on the response parameters are reported.

## 2. Design of Experiment and Material Selection

The material for the workpiece was aluminium 6061-T6. Aluminium is the third most plentiful element on earth [52] and is also widely used in architecture, transportation, and the food industry [53]. It is also widely used in the aerospace industry due to its high strength to weight ratio [54]. The motivation behind choosing this material for the research is to improve the surface quality and to reduce the energy consumption during the machining of this essential material. The chemical composition and mechanical properties of the material used are shown in Table 1 and Table 2, respectively.

The experiments in this study were designed by employing the Taguchi L9 array [56,57]. Each experiment was repeated thrice to obtain the accurate and precise results by the elimination of experimental variations. The feed per tooth $\left(fz\right),$ depth of cut $\left(a_{p}\right),$ number of inserts $\left(Z\right),$ and cutting speed $\left(Vc\right)$ were varied in three levels. The experimental plan is shown in Table 3.

**Figure 1:**
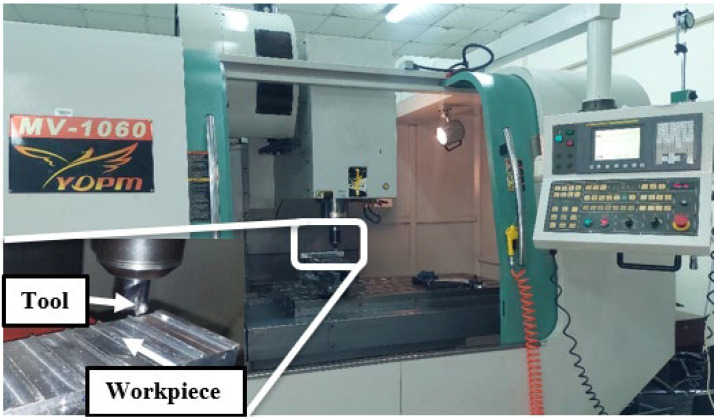
Machining setup.

The milling operation was carried out on an MV-1060 YDPM milling machine with a 25 mm diameter end milling cutter, as shown in Figure 1. A TIME^®^ 3110 roughness meter was used to measure surface roughness (μm). The meter uses an RC analogue filter and has a tracing length of 6 mm, with a speed of 1 mm/s. Every machining condition was repeated three times and each slot was cleaned with the air pressure and then with alcohol, and the surface roughness was measured thrice at different points, i.e., at the beginning of the slot, at the middle of the slot, and at the end of the slot. Burr width was measured using a metallurgical microscope (MEIJI Techno Co., Saitama, Japan, LTD Model: MT8530). It is a metallurgical microscope with an Infinity Corrected optical system F-200MM and a vertical Köhler illuminator with an infinity tube lens, a focal length 200 mm, and a 12 V 50 W halogen lamp. Energy consumption was measured using a three-phase power analyser (YOKOGAWA Model: CW240).

The material for the workpiece was aluminium 6061-T6. The selection of the cutting tool and insert was made using the Sandvik catalogue. The specifications of the tool holder, inserts, and cutting tool are shown in Table 4. It has been reported by previous researchers that tool wear during the machining process depends on the machining parameters [58]. Similarly, the tool wear can increase the energy consumption during machining [59,60]. To avoid the effect of tool wear on the energy consumption, a fresh insert was used for each cut. The specific cutting energy is defined as the energy consumed in removing a unit volume of material. It is calculated by Equation (1), where *MRR* is the material removal rate, and *P_cut_* is the amount of power consumed during the material removal, as shown in Equation (2).

(1) $$SCE=\frac{P_{cut}}{MRR}$$

(2) $$P_{cut}=P_{actual}-P_{air}$$

where *P_actual_* is the amount of power consumed during the air cut. In the air cut, the tool does not engage with the workpiece; however, it moves with the actual machining parameters. This includes the amount of power consumed during the complete motion of the tool, the lubrication system, and the illumination system. *P_cut_* is the amount of power consumed during the cutting or material removal process. In the recently published literature, specific cutting energy is considered a more authentic indicator for energy consumption [5,61,62]. R390-11 T3 02E-KM H13A of Sandvik was used on two different milling cutters, i.e., R390-0.25B25-11M and R390-028B25-11L. These cutters can mount 1, 2 and 3 inserts. The R390-0.25B25-11L was used to mount 1 and 2 inserts, whereas 3 inserts were used with the R390-025B25-11M milling cutter.

Specific cutting energy, burr width, and surface finish were investigated as a function of the machining parameters using ANOVA at a 95% confidence level (significance threshold of 0.05). The factors with a *p*-value lower than 0.05 were considered significant. The best and worst machining conditions are highlighted using the main effect plots, and the experiments for confirmation were performed twice on the best and worst machining conditions for optimising the individual response parameters.

## 3. Results and Discussion

A main effect plot and an analysis of variance for each machining parameter were used for the analysis. The effects of the input parameters on the individual output responses are discussed below.

### 3.1. Effects of Machining Parameters in Surface Roughness

**Figure 2:**
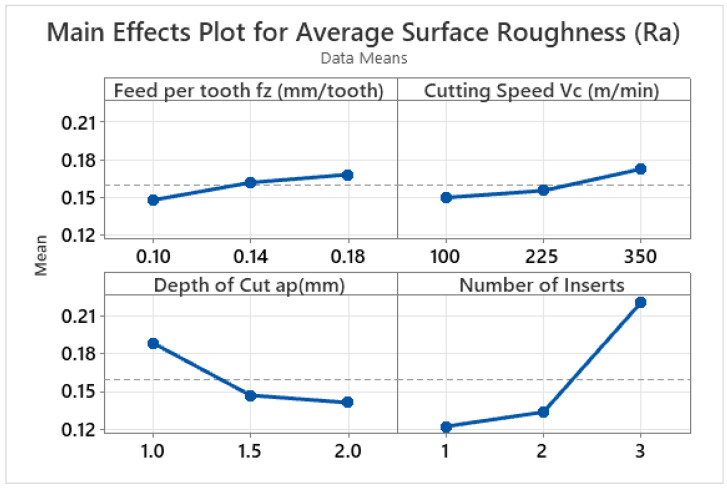
Main effect plots for average surface roughness (μm).

The effects of the varying machining parameters and the significance of each machining parameter are depicted by the main effect plots and analysis of variance in Figure 2 and Table 5, respectively. Figure 2 shows that with the increase in cutting speed, the number of inserts, and the feed per tooth, a rise in surface roughness is observed. On the other hand, the increase in the depth of cut results in a better surface finish. The increase in feed per tooth and the number of inserts increases the surface roughness, and this happens because the chip volume increases with the higher feed per tooth and the number of inserts. The higher chip volume is responsible for the higher surface roughness. The increase in cutting speed increases the temperature of the work piece [63]. As aluminium has relatively low melting point as compared to steel and other ferrous metals, the materials start to stick to the inserts at higher cutting speeds within the low-speed machining range. This phenomenon is less prominent than when machining ferrous alloys. Due to this reason, the inserts start to get blunt as a built-up edge is formed on the cutting edge of the insert; hence, it also increases the surface roughness. A similar pattern of surface roughness on steel [64,65] and on similar material has been reported earlier as well [8,13,66]. In addition to this, the higher cutting forces are also responsible for the higher surface roughness, and increasing the cutting speed increases the amount of cutting forces [67]. Furthermore, the increase in cutting speed also increases the vibration amplitude, due to which a higher surface roughness is generated [68]. It is pertinent to mention here that during high-speed machining, an increase in cutting speed also increases the surface quality; however, at low speed or a conventional machining range, the build-up edge is formed (especially for the metals with a lower melting point), which reduces the surface quality. As the specific cutting energy was found to decrease with the increasing depth of cut, the cutting forces also decreased with both the specific cutting energy and the cutting speed [69,70]. Due to the decreasing cutting forces, less elastoplastic deformation takes place, leading to improved surface roughness [43]. The reduction in SCE with the increase in the depth of cut is an indicator that the cutting forces are also reduced, and hence, a better surface finish is achieved with the increase in the depth of cut for this design of experiment [66]. The increase in the number of inserts increases the number of passes on the workpiece [71], and the vibrations are also increased. Both lead to higher surface roughness. A similar trend of increased surface roughness with the increase in the number of cutting edges on titanium alloy [72] and aluminium alloy 6061-T6 [73] were reported earlier as well.

An ANOVA for surface roughness with respect to the input parameters was carried out, and the results are displayed in Table 5. The depth of cut and the number of inserts were found to be significant input parameters with contribution ratios of 15.85% and 68.74%, respectively.

In addition, the cutting speed and feed per tooth were found to be insignificant members, as is indicated by their lower *p* value (higher than 0.05).

### 3.2. Effects of Machining Parameters on Specific Cutting Energy

**Figure 3:**
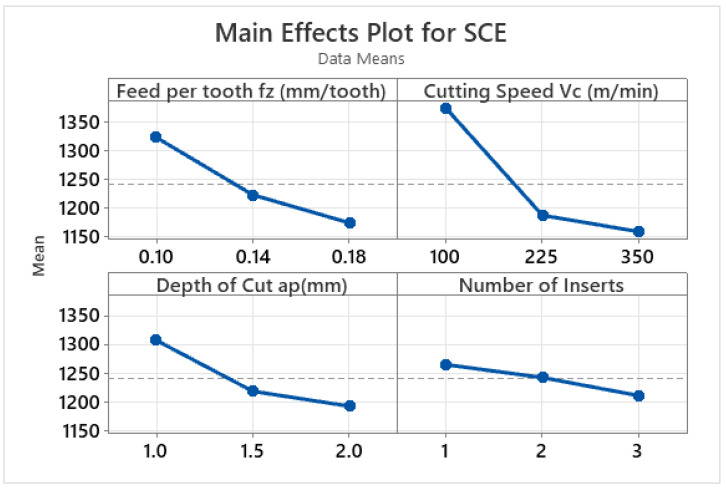
Main effect plots for specific cutting energy (J/cm^3^).

The effects of the varying machining parameters and the significance of each machining parameter are depicted by the main effect plots and analysis of variance in Figure 3 and Table 6, respectively. From Figure 3, the reduction in specific cutting energy is prominent with the increase in the values of all four machining parameters. The increase in the process parameters also increases the material removal rate and hence reduces the specific cutting energy consumption. In addition to this, the higher values of the process parameters also increase the temperature, which results in the softening of the material and hence reduces the specific cutting energy [29,31,74].

When considering the ANOVA results for the specific cutting energy, Table 6 shows that all the machining parameters had a significant impact on the specific cutting energy. In particular, cutting speed had the highest contribution ratio of 55%, whereas feed per tooth had a contribution ratio of 23%.

### 3.3. Effect of Machining Parameters on Burr Width

During the analysis of the experimental results, it was observed that smaller sized burrs were produced on the down-milling side in comparison to the up-milling side of the work piece; this is due to the fact that if a material deforms in the direction of the force it produces smaller sized burrs [10]. This is also due to the fact that a higher velocity of the localized cutting edge produces smaller sized burrs [75]. A similar pattern has been reported previously [76,77].

**Figure 4:**
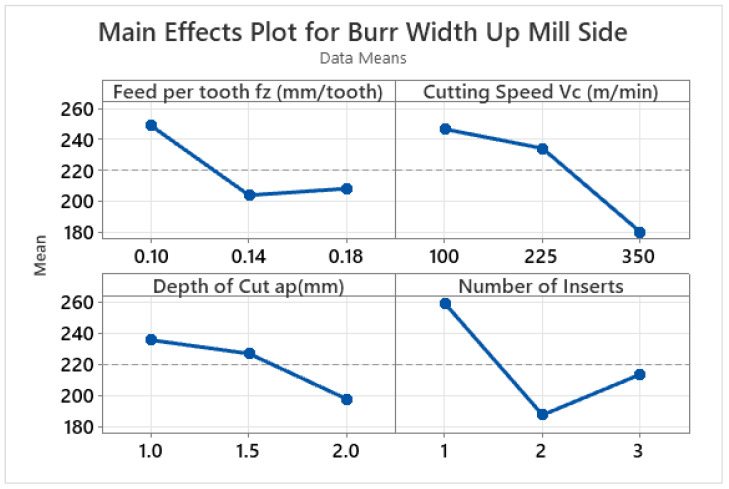
Main effect plots for burr width on up-milling side (μm).

**Figure 5:**
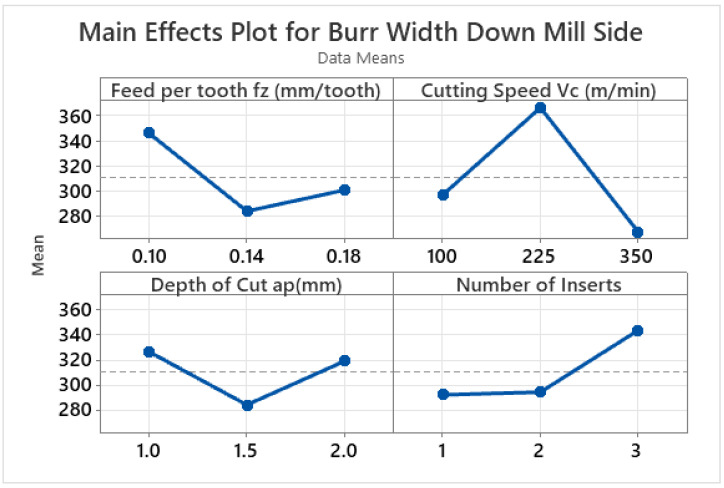
Main effect plots for burr width on down-milling side (μm).

On the up-milling side, the burr width first declines and then subsequently grows with the increase in the feed per tooth and number of inserts, whereas the burr width reduces with the increase in the depth of cut and cutting speed, as illustrated in Figure 4. The main effect plot of the down-milling side (Figure 5) shows that the burr width initially reduces and then increases as the feed per tooth and depth of cut increase. Conversely, the burr width initially increases and eventually reduces with the increase in cutting speed. In the case of the number of inserts, the burr width increases throughout the range. In the case of the ductile materials, the larger value of the depth of cut produces a smaller amount of tensile stress on the chips which are about to detach from the work piece. Due to this smaller amount of stress, the smaller sized burrs are produced at higher depths of cut [78]. At higher values of the number of inserts and cutting speed, the ploughing effect is more significant and hence also increases the burr size [12,78]. A very limited amount of literature has been published on burr formation during macro milling; however, keeping in mind the published literature, it can be said that no consistent behaviour of burr size can be predicted [9,19,79,80].

The results of the ANOVA for the up-milling side of the workpiece are given in Table 7. It is pertinent to mention that all four input variables have a substantial impact on burr width on the up-milling side, with the number of inserts having the highest contribution ratio of 35%. The second important parameter is observed to be the cutting speed, with a contribution ratio of 32.82%.

Similarly, in the case of the down-milling side, the highest contribution ratio of 47.98% was achieved by the cutting speed, as depicted by the ANOVA results given in Table 8. The contribution ratio of the feed per tooth and the number of inserts was determined to be 19.30% and 15.71%, respectively.

From the results of the analysis of variance for the down-milling side of the workpiece (shown in Table 8), it is observed that the four varied machining parameters play a significant role in affecting burr width as the *p*-value for the machining parameters is less than 0.05. The cutting speed contributes the most to the contribution ratio, followed by feed per tooth, number of inserts, and depth of cut.

The best and worse machining parameters for burr width on the up- and down-milling sides are noted from the main effect plot; the experiments were twice repeated on these machining parameters. The results for the best and worst machining conditions for the burr width on the up- and down-milling sides are shown in Table 9.

### 3.4. Validation of Results

The focus of this experimental study is to reduce surface roughness, specific cutting energy, and burr width during the milling of aluminium alloy Al 6061-T6. After the identification of the significant machining parameters and the analysis of variance, the best and worst machining conditions were identified, as given in Table 9. Afterwards, an experimental validation of the current design of experiments was conducted by machining at the identified best and worst combination of input parameters for each individual output response. The results, as given in Table 9, confirm the validity of the experimental runs.

**Figure 6:**
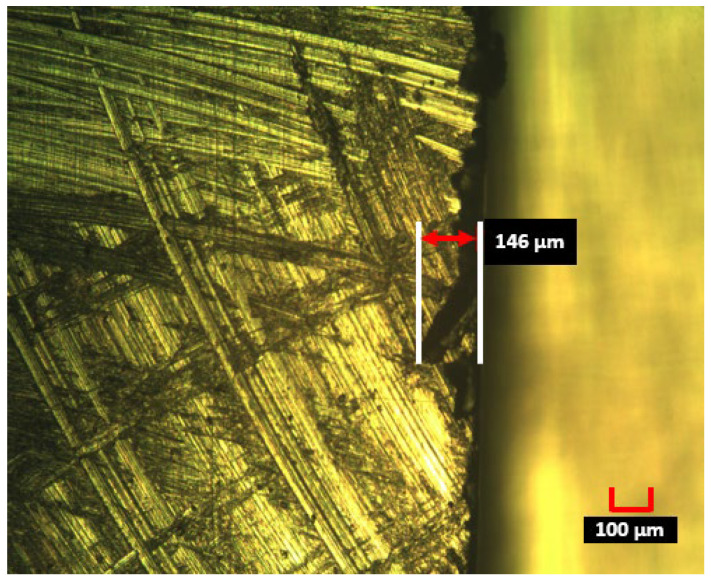
Minimum burr width noted on up-milling side of the workpiece.

**Figure 7:**
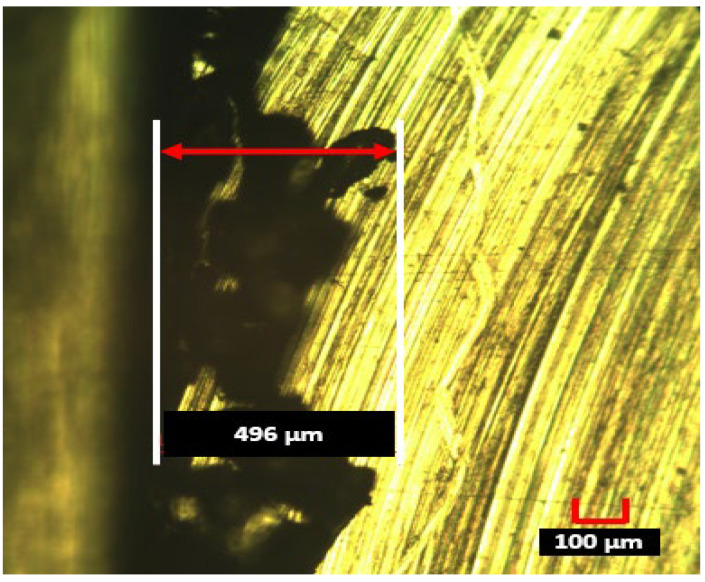
Maximum burr width noted on down-milling side of the workpiece.

The results of the best burr width on the up-milling side and the worst burr width on the down-milling side are shown in Figure 6 and Figure 7, respectively.

## 4. Conclusions

The current experimental study was designed to investigate the effect of individual machining parameters on the response parameters. The statistical analysis, including main effect plots and ANOVA, was employed to identify the effects of the input parameters and to determine their contribution ratios. The following conclusions were drawn during the conducting of this study.

1. The four machining parameters that varied in this study played a significant role in affecting average surface roughness, specific cutting energy, and burr width. The number of inserts was noted to be the most significant parameter in affecting burr width on the up-milling side (contribution ratio 35%) and the average surface roughness (68.74%), whereas the cutting speed was noted to be the most significant parameter in affecting burr width on the down-milling side (47.98%) and specific cutting energy consumption (55.40%).
2. Surface finish may be enhanced by reducing the number of inserts, decreasing the feed per tooth and cutting speed, and increasing the depth of cut. Hence, smaller values of the number of inserts, cutting speed, and feed per tooth and higher values of depth of cut should be used during any milling operation to obtain minimum surface roughness. Keeping in mind the published literature, the pattern of surface roughness by varying machining parameters is different in the high-speed conventional machining range.
3. Specific cutting energy may be minimized by increasing the feed per tooth, cutting speed, depth of cut, and number of inserts. Consequently, to minimize the specific cutting energy, larger values of the given machining parameters may preferably be used.
4. Burr width was greater on the down-milling side as compared to the up-milling side of the workpiece for the same cutting conditions. The burr width on the up-milling side was decreased as the cutting speed and depth of cut increased, whereas the burr width on the down-milling side was decreased as the number of inserts decreased.
5. During the validation experiments, a minimum burr width of 146 μm on the up-milling side was observed. Similarly, the highest burr width of 496 μm was recorded on the down-milling side.
6. By selection of the appropriate machining parameters, it is possible to reduce surface roughness, specific cutting energy, and burr width. However, it is not possible to achieve a minimum surface finish, specific cutting energy, and burr width simultaneously. This points to a need for multi-objective optimization to set a trade-off between the four output parameters in future research. In addition to this formation of an energy map, considering the most significant machining parameters would be a great research contribution towards sustainable machining. Nevertheless, maps of the response parameters for different materials by the varying machining conditions will be helpful in obtaining the desired outputs.

## Figures and Tables

**Table 1:** Chemical composition of aluminium 6061-T6 [55].

	Si	Fe	Cu	Mn	Mg	Cr	Zn	Ti	Al
%	0.62	0.22	0.29	0.07	1.1	0.18	0.01	0.01	~Bal

**Table 2:** Mechanical properties of aluminium 6061-T6 [55].

Tensile Strength (MPa)	Yield Strength (MPa)	Elongation %	Hardness (HV)
280–300	250–260	12.0–14.0	101–108

**Table 3:** Machining parameters and their levels used in this study.

Depth of Cut (mm)	Cutting Speed (m/min)	Feed per Tooth (mm/Tooth)	Number of Inserts
1	100	0.1	1
1.5	225	0.14	2
2	350	0.18	3

**Table 4:** Tool holder, end miller cutter, and insert specifications.

Specifications	Descriptions
Tool holder	WALTER A170M.063.080.25
End mill cutter	R390-0.25B25-11M and R390-028B25-11L
Insert	R390-11 T3 02E-KM H13A
Tool diameter	25 mm
Maximum cutting speed (m/min) of insert	1000
Feed per tooth (mm/tooth)	0.08–0.18

**Table 5:** ANOVA results for average surface roughness.

Source	DF	Seq SS	Contribution	Adj SS	Adj MS	F-Value	*p*-Value
Feed per Tooth fz (mm/tooth)	2	0.001935	2.54%	0.001935	0.000967	2.40	0.119
Cutting Speed Vc (m/min)	2	0.002539	3.33%	0.002539	0.001269	3.14	0.067
Depth of Cut ap (mm)	2	0.012079	15.85%	0.012079	0.006040	14.96	0.001
Number of Inserts	2	0.052385	68.74%	0.052385	0.026192	64.88	0.001
Error	18	0.007267	9.54%	0.007267	0.000404		
Total	26	0.076204	100%				

**Table 6:** ANOVA results for specific cutting energy.

Source	DF	Seq SS	Contribution	Adj SS	Adj MS	F-Value	*p*-Value
Feed per Tooth fz (mm/tooth)	2	102,842	23.47%	102,842	51,421	61.95	0.001
Cutting Speed Vc (m/min)	2	242,792	55.40%	242,792	121,396	146.24	0.001
Depth of Cut ap (mm)	2	64,633	14.75%	64,633	32,317	38.93	0.001
Number of Inserts	2	13,057	2.98%	13,057	6528	7.86	0.004
Error	18	14,942	3.41%	14,942	830		
Total	26	438,266	100.00%				

**Table 7:** ANOVA results for burr width on up-milling side.

Source	DF	Seq SS	Contribution	Adj SS	Adj MS	F-Value	*p*-Value
Feed per Tooth fz (mm/tooth)	2	10,958	16.24%	10,958	5479.1	27.21	0.001
Cutting Speed Vc (m/min)	2	22,146	32.82%	22,146	11,072.9	54.99	0.001
Depth of Cut ap (mm)	2	7117	10.55%	7117	3558.3	17.67	0.001
Number of Inserts	2	23,635	35.02%	23,635	11,817.3	58.69	0.001
Error	18	3625	5.37%	3625	201.4		
Total	26	67,480	100.00%				

**Table 8:** ANOVA results for burr width on down-milling side.

Source	DF	Seq SS	Contribution	Adj SS	Adj MS	F-Value	*p*-Value
Feed per Tooth fz (mm/tooth)	2	18,637	19.30%	18,637	9318.4	23.33	0.001
Cutting Speed Vc (m/min)	2	46,329	47.98%	46,329	23,164.4	58.01	0.001
Depth of Cut ap (mm)	2	9240	9.57%	9240	4,620.2	11.57	0.001
Number of Inserts	2	15,172	15.71%	15,172	7585.8	19	0.001
Error	18	7188	7.44%	7188	399.3		
Total	26	96,566	100.00%				

**Table 9:** Best and worst machining conditions for recorded responses.

Responses		Machining Parameters				Results
		Feed Per Tooth (mm/Tooth)	Cutting Speed (m/min)	Depth of Cut (mm)	Number of Inserts	
Average Surface roughness (μm)	Best	0.1	100	2	1	0.10
Average Surface roughness (μm)	Worst	0.18	350	1	3	0.27
Specific Cutting Energy (J/cm^3^)	Best	0.18	350	2	3	966.99
Specific Cutting Energy (J/cm^3^)	Worst	0.1	100	1	1	1548.95
Burr width on up-milling side (μm)	Best	0.14	350	2	2	153
Burr width on up-milling side (μm)	Worst	0.1	100	1	1	344
Burr width on down-milling side (μm)	Best	0.14	350	1.5	1	139
Burr width on down-milling side (μm)	Worst	0.1	225	1	3	485

## Data Availability

The data presented in this study are available upon request from the corresponding author.
